# PC 12 Pheochromocytoma Cell Response to Super High Frequency Terahertz Radiation from Synchrotron Source

**DOI:** 10.3390/cancers11020162

**Published:** 2019-01-31

**Authors:** Palalle G. Tharushi Perera, Dominique R. T. Appadoo, Samuel Cheeseman, Jason V. Wandiyanto, Denver Linklater, Chaitali Dekiwadia, Vi Khanh Truong, Mark J. Tobin, Jitraporn Vongsvivut, Olha Bazaka, Kateryna Bazaka, Rodney J. Croft, Russell J. Crawford, Elena P. Ivanova

**Affiliations:** 1 Faculty Science, Engineering and Technology, Swinburne University of Technology, P.O. Box 218, Hawthorn, VIC 3122, Australia; pgperera@swin.edu.au (P.G.T.P.); jwandiyanto@swin.edu.au (J.V.W.); 2 THz/Far-Infrared Beamline, Australian Synchrotron, Clayton, VIC 3168, Australia; Dominique.APPADOO@ansto.gov.au (D.R.T.A.); tobinm@ansto.gov.au (M.J.T.); jitrapov@ansto.gov.au (J.V.); 3 School of Science, RMIT University, P.O. Box 2476, Melbourne, VIC 3001, Australia; s3741431@student.rmit.edu.au (S.C.); vi.khanh.truong@rmit.edu.au (V.K.T.); olga.bazaka@jcu.edu.au (O.B.); russell.crawford@rmit.edu.au (R.J.C.); 4 Centre for Micro-Photonics, Swinburne University of Technology, Hawthorn, VIC 3122, Australia; dlinklater@swin.edu.au; 5 RMIT Microscopy and Microanalysis Facility, College of Science, Engineering and Health, RMIT University, P.O. Box 2476, Melbourne, VIC 3001, Australia; chaitali.dekiwadia@rmit.edu.au; 6 School of Chemistry, Physics, Mechanical Engineering, Queensland University of Technology, Brisbane, QLD 4000, Australia; kateryna.bazaka@qut.edu.au; 7 School of Psychology, Illawarra Health and Medical Research Institute, University of Wollongong, Wollongong, NSW 2522, Australia; rcroft@uow.edu.au

**Keywords:** terahertz exposure, cell viability, PC 12 neuronal cells, super high frequency electromagnetic radiation

## Abstract

High frequency (HF) electromagnetic fields (EMFs) have been widely used in many wireless communication devices, yet within the terahertz (THz) range, their effects on biological systems are poorly understood. In this study, electromagnetic radiation in the range of 0.3–19.5 × 10^12^ Hz, generated using a synchrotron light source, was used to investigate the response of PC 12 neuron-like pheochromocytoma cells to THz irradiation. The PC 12 cells remained viable and physiologically healthy, as confirmed by a panel of biological assays; however, exposure to THz radiation for 10 min at 25.2 ± 0.4 °C was sufficient to induce a temporary increase in their cell membrane permeability. High-resolution transmission electron microscopy (TEM) confirmed cell membrane permeabilization via visualisation of the translocation of silica nanospheres (*d* = 23.5 ± 0.2 nm) and their clusters (*d* = 63 nm) into the PC 12 cells. Analysis of scanning electron microscopy (SEM) micrographs revealed the formation of atypically large (up to 1 µm) blebs on the surface of PC 12 cells when exposed to THz radiation. Long-term analysis showed no substantial differences in metabolic activity between the PC 12 cells exposed to THz radiation and untreated cells; however, a higher population of the THz-treated PC 12 cells responded to the nerve growth factor (NGF) by extending longer neurites (up to 0–20 µm) compared to the untreated PC12 cells (up to 20 µm). These findings present implications for the development of nanoparticle-mediated drug delivery and gene therapy strategies since THz irradiation can promote nanoparticle uptake by cells without causing apoptosis, necrosis or physiological damage, as well as provide a deeper fundamental insight into the biological effects of environmental exposure of cells to electromagnetic radiation of super high frequencies.

## 1. Introduction

There has been recent growing interest, across a broad range of scientific fields, in the application of continuous-wave and pulsed electromagnetic radiation in the terahertz (THz) frequency range due to their potential for use in detection, imaging and communications technologies. The THz frequency range covers the portion of the electromagnetic spectrum that falls between microwaves and the infrared domains and is generally defined as including frequencies between 0.3–10 THz [1]. THz photons are not sufficiently energetic to break chemical bonds or cause ionisation of atoms or molecules [2]. The ability of THz radiation to penetrate a wide variety of non-conducting materials, e.g., clothing, cardboard and plastic, while being strongly attenuated in metals or water, presents a number of unique advantages for applications such as non-destructive imaging in biomedical applications, non-invasive national security and packaged goods inspection, and remote environmental sensing [3,4,5]. THz radiation is also used for the time-resolved spectroscopic investigation of multibody systems and difficult-to-probe materials, such as complex nanostructures and foams, to reveal their fundamental properties, including chemical composition, density, defect locations, and their technological potential [2]. Upon interaction with molecules in a material, THz waves can induce low-energy excitations, such as phonon, electronic interband, impurity-related and intraexcitonic transitions and intersubband excitations in solids, as well as intermolecular vibrations and translations in liquids [2]. These excitations modulate the intensity and phase of the THz wave. The nature of these weak material bond vibrations and deformations correspond to specific chemical and physical properties of the material. The high coherence characteristics (i.e., high spatial and strong temporal properties) of THz radiation also enhances absorption coefficients and refractive indices of samples [6].

An important application of THz time-domain spectroscopy (THz-TDS) and imaging is the accurate detection and diagnosis of skin, breast, colon and liver cancer both, in vivo and ex vivo [3]. For example, when used on freshly excised tissues, THz-TDS operated in reflection mode was able to successfully differentiate between regions of cancerous, normal and pathologically changed tissue, with clearly distinguishable waveforms of reflected signals [7]. Similar results have been obtained on excised paraffin-embedded human breast tissue using THz transmission and reflection imaging, with the reflection mode providing better resolution and sharper margins between cancerous, fibroglandular and fatty tissue regions, and the absorption mode being less sensitive to variations in tissue thickness [8].

The viability of in vivo applications of THz imaging for the detection of tissue hydration, corneal pathologies, skin burns and cancers depends upon the principle that terahertz photons are not energetic enough to cause lasting perturbations in biological materials, or to cause DNA damage [9]. Whether this is indeed the case, however, remains a subject of debate as studies are limited by the scarcity of efficient THz generating sources and sensitive detectors [6]. For example, human dermal fibroblasts exposed to continuous THz radiation from an optically pumped molecular gas THz laser source (2.52 THz, 227 mW/cm^2^) for up to 80 min were shown to be 90% viable [10,11,12]. When exposed to the narrow-band THz radiation (2.3 THz, 1.4 W/cm^2^), human embryonic stem cells (hESCs) showed no structural chromosomal aberrations or difference in cell morphology when compared to the untreated control, and only some minor upregulations of mitochondria-related genes were reported [1]. Similarly, primary human keratinocytes exposed to THz radiation (0.2–3.0 THz, 0.45 J/cm^2^) for 30 min showed only minor donor-specific inhibition or stimulation of cell activity [13]. Other groups, however, have suggested that THz radiation, and thus generated electric fields, may affect complex molecular processes involved in gene expression and DNA replication by inducing a large dipole moment in DNA [5] by producing vibrational modes in the hydrogen bonds in nucleobases [14] and causing spatially localised unbinding of the strands in DNA helices [15]. Using mouse mesenchymal stem cells, heterogenic changes in gene expression were shown to be both irradiation and cell specific, i.e., the activation and repression of genes were found to be dependent on duration and type of THz source (e.g., pulsed 10 THz vs. continuous wave 2.52 THz, 1–3 mW/cm^2^), as well as on the stage of stem cell differentiation [16].

In addition to modulating gene expression, THz exposure was also suggested to affect the cell membrane. It was previously reported that protrusions of the membrane on the cell surfaces and a decrease in the membrane potential was detected in 12–15% mollusc neuron cells irradiated at 30 mW/cm^2^ for 1 min [17]. Nevertheless, little is known about the specific effects of THz radiation on cell membrane and intracellular metabolic activity.

This study aims to fill this significant knowledge gap by investigating the possible effects of THz radiation (0.3–19.5 THz), generated using a synchrotron light source, on a model mammalian cell line, namely PC 12 pheochromocytoma cells. PC12 cell lines are derived from a pheochromocytoma of the rat adrenal medulla, a mixture of neuroblastic cells and eosinophilic cells [18,19]. Pheochromocytoma was chosen as a model cell line due to the large amount of background knowledge with respect to its proliferation and differentiation. The results of this study aim to provide a deeper fundamental insight into the biological effects of the environmental exposure of cells to electromagnetic radiation as well as to investigate whether THz radiation can induce transient cell membrane permeabilisation and promote nanoparticle uptake by cells without causing apoptosis, necrosis or physiological damage. The significance of the research is that it highlights the possibility of using THz irradiation of mammalian cells as a strategy to enhance nanoparticle-mediated drug delivery in the treatment of cancers [20] and gene therapy [21], similar to 18 GHz radiation [22]. The research investigates the efficiency of the technique in the context of delivering nanospheres into the cell and the ability of nanospheres to pass the cell membrane. The use of EMFs in delivering nanospheres can be promising in the case of metastatic pheochromocytoma. It is critical to study the specific outcomes including PC 12 cell proliferation and differentiation in during and after THz radiation for clinical applications.

## 2. Results and Discussion

The PC 12 cells were exposed to THz radiation at frequencies ranging from 0.3 to 19.5 THz for a period of 10 min. During the THz radiation exposure, the average temperature of the sample was recorded as 25.24 ± 0.37 °C. Assuming no power loss to the surrounding medium, the PC 12 cells were considered to have absorbed all the energy delivered to the sample. The increase in temperature measured in five different locations of the sample was in the range of only ± 0.37 °C due to the low power of the THz beam.

**Figure 1:**
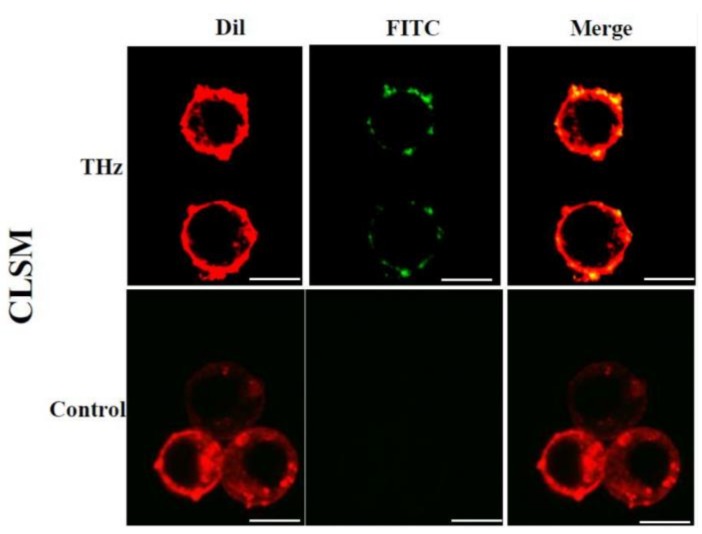

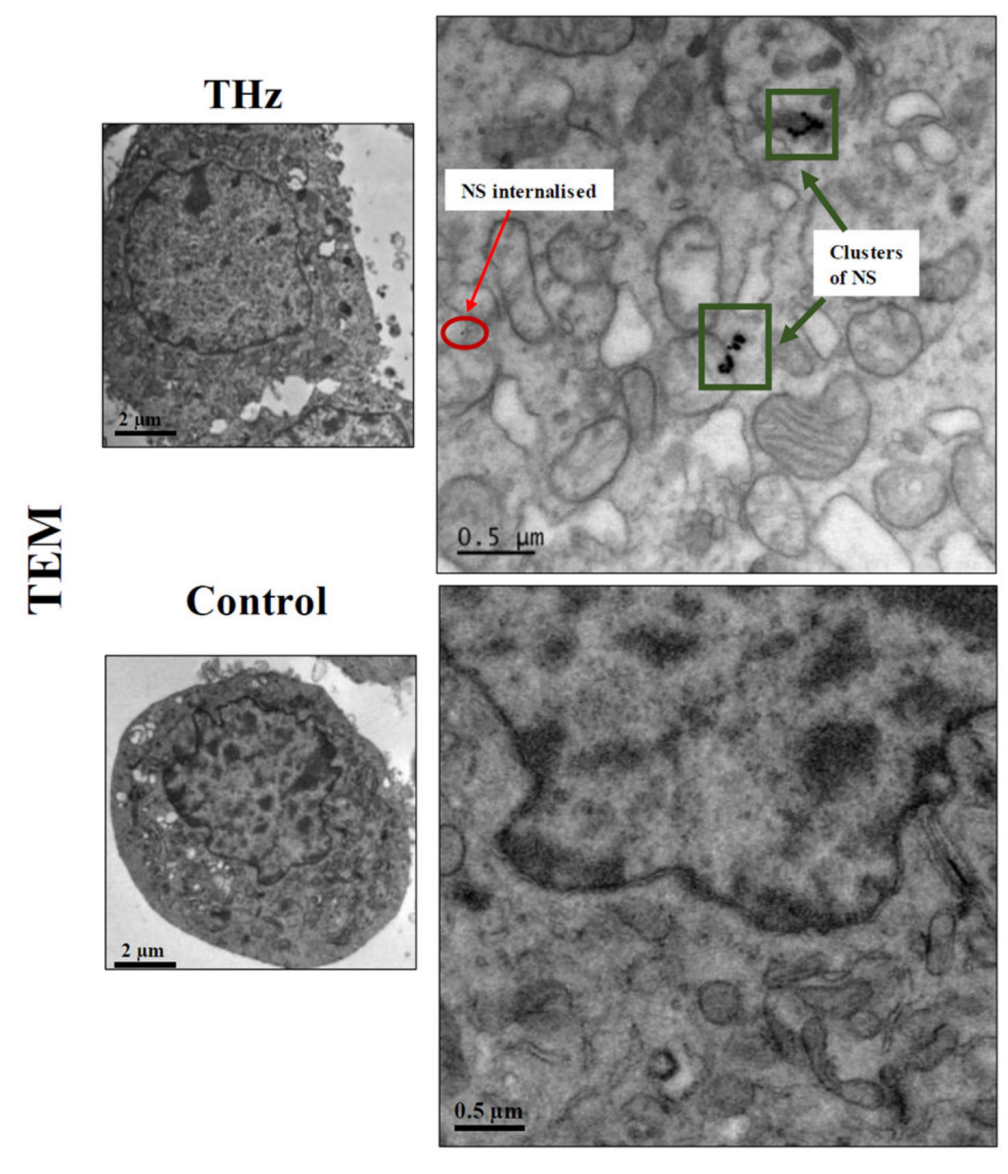
Nanosphere internalisation of PC 12 cells following a 10 min exposure of THz radiation. Confocal laser scanning microscopy (CLSM; top row) images illustrate the uptake of silica nanospheres (FITC) by the THz treated cells whereas the untreated control does not exhibit any nanosphere uptake. No signal was detected in the FITC channel for the untreated cells. Scale bar is 5 μm. Thin sliced transmission electron microscopy (TEM) micrographs confirm silica nanospheres (NS) being internalised by the PC 12 cells (red arrow; bottom). Nanospheres are also seen lining the cell membrane whereas no nanosphere internalisation was observed in the untreated control cells. Scale bar is 1 μm.

**Figure 2:**
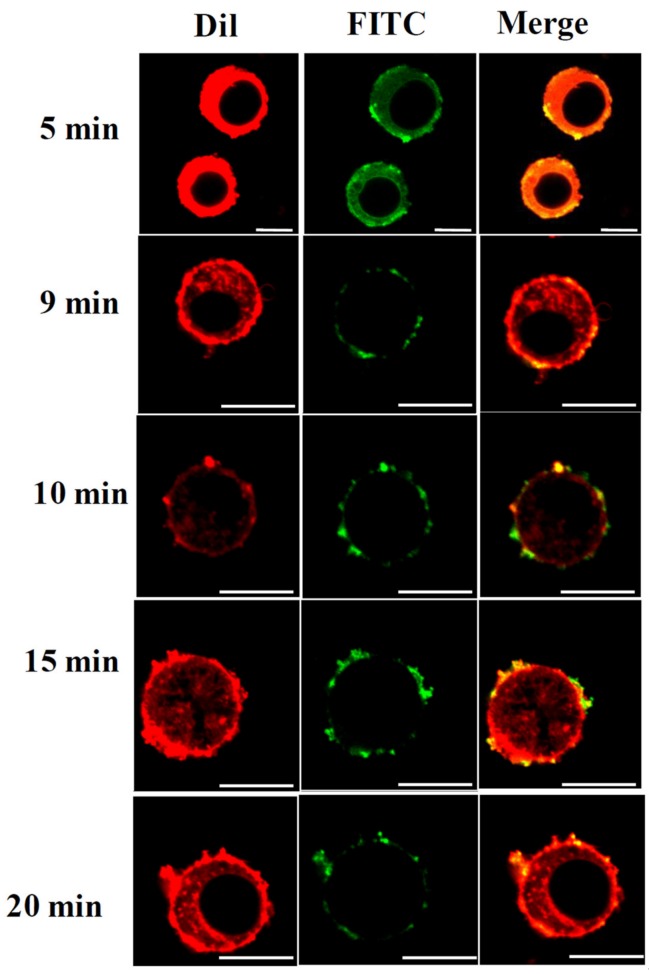
Duration of PC 12 cell permeability following exposure to THz radiation. CLSM micrographs showing fluorescent silica nanospheres being internalised by PC 12 cells, after 5, 9, 10, 15, and 20 min following exposure. PC 12 cells were permeable for up to 20 min after being exposed to THz radiation. Scale bar is 10 μm.

Here, exposure to electromagnetic fields in the THz range triggered an increase in cell membrane permeability of PC 12 cells. Increased permeability was evidenced by an increased internalisation of silica nanospheres (*d* = 23.5 nm) and their clusters of ≈63.9 nm by the cells, as compared to the untreated control cells (Figure 1 and Figure 2). The uptake of the FITC-labelled silica nanospheres, which can be seen embedded in the cellular membrane was confirmed using confocal laser scanning microscopy (CLSM) and TEM (Figure 1). Visual examination of the TEM images revealed that nanospheres were present on the lining of the cell membrane, as well as clusters being observed in the cytoplasm, external to intracellular vesicles (Figure 1). Approximately 95% of the treated PC 12 cells were able to uptake the nanospheres following THz radiation exposure, while the nanospheres uptake by untreated PC 12 cells was negligible (4–5%). Silica nanospheres have an innate propensity to form clusters in working solution. In our recent work, we confirmed that the majority of the nanospheres in working solution appeared to be in clusters of 3 or 4 nanospheres with the average size of the majority of clusters being 63.9 nm [22]. Single nanospheres represented less than 10% of the total nanospheres present in the working solution. The results of this study showed that subsequent to THz radiation exposure, PC 12 cells were able to internalise both individual nanospheres (red insets) and their clusters (green insets), which were then located inside the cell cytoplasm (Figure 1). The quantification of the nanospheres uptake by a single cell revealed that THz treated PC 12 cell was able to internalize 73 ± 9.8 clusters of the nanospheres, and approximately 5 ± 3.0 single nanospheres. The untreated control cells did not demonstrate any internalisation of nanospheres. Similar results were observed using electromagnetic radiation at a frequency of 18 GHz where a reversible increase in membrane permeability was observed in PC 12 cells [22], as well as in different Gram-negative and Gram-positive bacterial species: *Planococcus maritimus* KMM 3738, *Staphylococcus aureus* CIP65.8^T^, *S. aureus* ATCC 25923, *S. epidermidis* ATCC 14990^T^, *Escherichia coli* ATCC 15034, yeast (*Saccharomyces cerevisiae* ATCC 287) and red blood cells [23,24].

The internalisation of the nanospheres by the cells was also quantified using the permeability co-efficient [22,25], which depended on the fluorescence intensity detected from the fluorescent silica nanospheres. The concentration of the nanospheres in the THz radiation-exposed suspension was calculated to be 0.05 μg/mL, which corresponds to 4.6 × 10^7^ nanospheres. By dividing the mass of a single nanosphere, the number of internalized nanospheres was 2.7 × 10^12^. Since the cell concentration was adjusted to 60,000 cells/mL, the number of internalized nanospheres per PC 12 cell was calculated to be 4.6 × 10^7^ nanospheres. Notably, exposure of PC 12 cells to an 18 GHz EMF allowed for the internalization of 2.8 × 10^6^ nanospheres [22]. The greater number of nanospheres that could be internalised by the THz radiation-exposed PC 12, in comparison to GHz irradiated PC 12 cells, could be due to the longer time of exposure (10 min) compared to that of the GHz radiation exposure (30 s) [22].

To understand the temporal change in cell permeability, the degree of nanosphere internalisation was evaluated at time points of 5, 9, 10, 15, and 20 min following THz irradiation. Our results suggest that it was possible for THz radiation-induced permeability in PC 12 cells to last for up to 20 min (Figure 2). This is considerably longer than that achieved using electromagnetic radiation at a frequency of 18 GHz [22]. The induced membrane permeability could be due to the strong dipole moments of the water molecules being induced as a result of the THz radiation exposure. It has been previously suggested that water molecules in the medium will strongly absorb the THz radiation, a process that induces molecular vibrations that, in turn, leads to highly localised increases in temperature that may affect biological systems [1,6].

**Figure 3:**
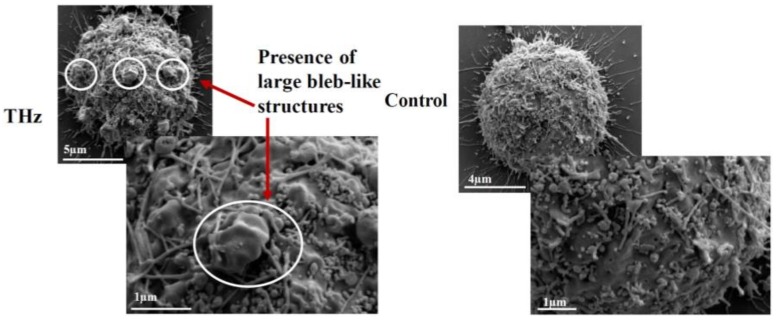
Scanning electron micrographs of PC 12 cells after 10 min of THz exposure. Following exposure to THz radiation, morphology analysis revealed significant bleb formation on the PC 12 cells (circled in white), whereas blebs were not present on the untreated PC 12 cells.

In addition to changes in membrane permeability, analysis of the PC 12 cell morphology using SEM revealed the formation of large (1–2 µm) bleb-like structures, or spherical membrane protrusions, on the surfaces of PC 12 cells exposed to THz radiation (Figure 3). Non-treated cells exhibited typical cell morphology with small blebs of 0.3–0.5 µm in size. Bleb formation is observed mainly during cell migration, cytokinesis, apoptosis or cell spreading [26,27]. The formation of blebs is initiated by the contractile actomysin cortex, which creates hydrostatic pressure in the cytoplasm, thus leading to herniation of the plasma membrane [26]. The cellular cortex consists of a thin meshwork of myosin, actin filaments and other proteins, and is able to exert pressure on the cell cytoplasm [26]. It is possible that electromagnetic fields with frequencies in the THz range may cause deformation of the cell membrane as a result of the pressure exerted on the cell cytoplasm. It is interesting to note that no bleb formation was detected in PC 12 cells following exposure to electromagnetic fields at a frequency of 18 GHz [22]. In a study conducted by Shamis et al. [24] using microwave radiation, cytoplasmic leakage was observed in *E. coli* following EMF exposure. No cytoplasmic leakage was observed in THz radiation-treated PC 12 cells, suggesting that PC 12 cells may form blebs in an effort to minimise intracellular changes induced by the THz radiation.

**Figure 4:**
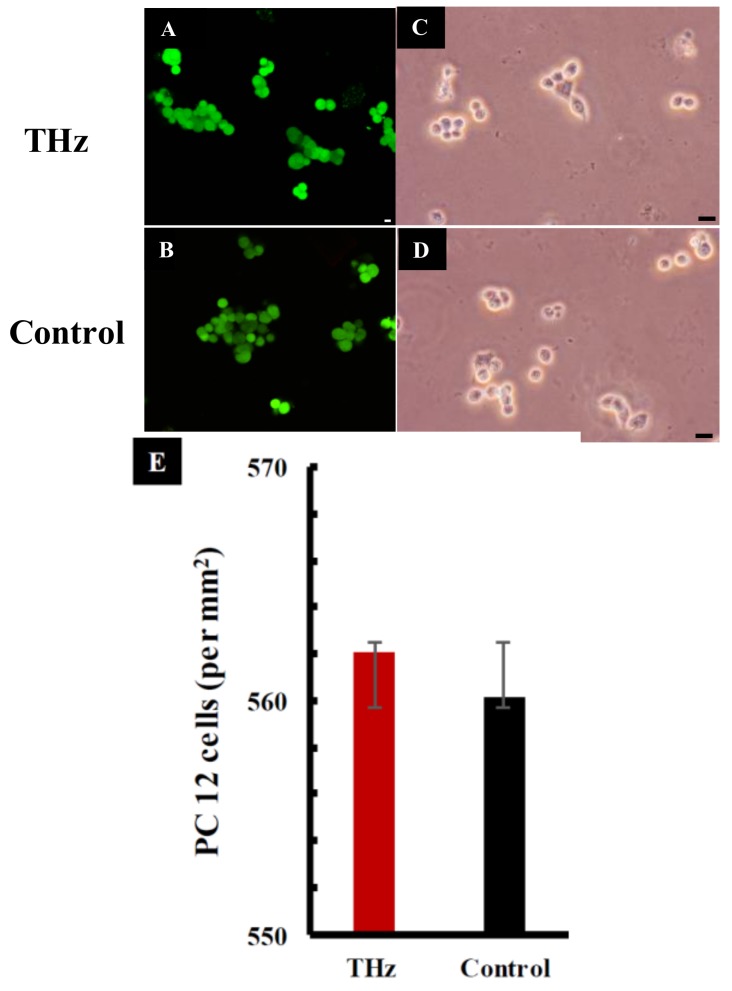
Physiological analysis of PC 12 cells after THz exposure of 10 min. (**A**) Confocal laser scanning images showing viable (green) PC 12 cells after being exposed to synchrotron source THz radiation for 10 min. (**B**) The untreated control cells. Scale bar is 5 μm. (**C**) Optical micrographs of PC 12 cells following exposure to THz radiation and (**D**) the untreated control. Scale bar is 10 μm. (**E**) Quantification of viable PC 12 cells following exposure to THz radiation. No significant changes in cell viability were detected between the THz treated and the control group (*p* = 0.977). Data are presented as mean ± SD and are representative of the three independent repeats.

The viability of the PC 12 cells was investigated using CLSM and phase contrast micrographs. These studies revealed that treated cells exhibited a similar viability to that of the untreated control cells following THz radiation exposure for a period of 10 min (Figure 4A,B). The cell number per mm^2^ was quantified and a subsequent statistical analysis did not reveal a statistically significant difference between the viability of cells in the THz-treated and control groups (*p* > 0.05). In a study performed by Antonopoulos et al. [28], human lymphocytes were incubated in the presence of high-frequency electromagnetic fields. No difference in the cell cycle progression was observed between the treated and control culture groups. Similarly, PC 12 cells exposed to 18 GHz EMFs did not show any changes in cell viability compared to that of the untreated control group, suggesting that the cells were able to remain viable after short-term exposure to EMFs of these types.

**Figure 5:**
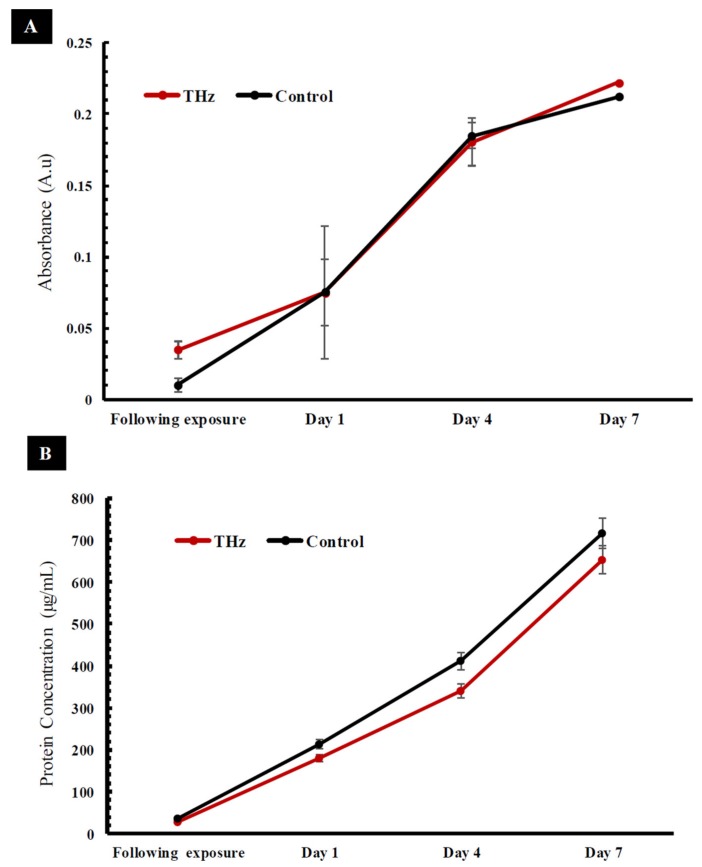
Term physiological analysis of PC 12 cells after THz exposure of 10 min. (**A**) Metabolic status (MTS) over the duration of 7 days and following exposure. The metabolic activity of THz treated PC 12 cells were similar to the untreated control following exposure (*p* = 0.803) after day 1 and no significant changes were observed over the course of 7 days. (**B**) The total protein concentration of the THz treated sample after exposure and over a 7-day period. The total protein content of the THz treated sample was relatively low in comparison to the control straight after exposure (*p* = 0.574) and over the course of 7 days. The statistical analysis of the results did not reveal a statistically significant difference (*p* > 0.05). Data are means ± standard deviation (SD) and representative of three independent repeats.

The metabolic activity of cells was analysed immediately after 10 min of exposure to THz radiation. Increased metabolic activity was detected in PC 12 cells exposed to THz radiation in comparison to the untreated control (*p* = 0.803) (Figure 4E). The mitochondrial activity of metabolically active cells resulted in the conversion of tetrazolium dye MTS (3-(4,5-dimethylthiazol-2-yl)-5-(3-carboxymethoxyphenyl)-2-(4-sulfophenyl)-2H-tetrazolium) (salt) into a soluble purple formazan [29]. The absorbance of formazan, recorded at 490 nm, is directly proportional to the cell metabolic activity. The reduction of MTS mainly takes place in the mitochondria, hence providing a measure of mitochondrial function [30]. Previous studies have shown that exposure of PC 12 cells to 18 GHz EMFs causes increased metabolic activity when compared to that of the untreated control cells [22]. An increased level of enzymatic activity of acetylcholinesterase (AChE) and a higher lactate dehydrogenase (LDH) enzyme activity in *E. coli* [31] was also reported in PC 12 cells exposed to 1.8 GHz radiation [32]. The metabolic activity of PC 12 cells was monitored every two days over a period of 7 days (Figure 5A) as PC 12 cells double every 36–48 h [33]. At day 1, the metabolic activity of the THz treated PC 12 cells and the untreated control cells exhibited a similar metabolic activity, and this trend remained constant over the 7-day observation period (Figure 5A) (the difference in respective absorbance values was found to be not statistically significant between the two experimental groups). These results suggest that super high frequency THz radiation exposure does cause an initial short-term effect on the PC 12 cells, but over a period of time, the metabolic activity of the cells returned to normal.

A bicinchoninic acid (BCA) assay was used to evaluate the total protein content of the PC 12 cells following exposure to THz radiation (Figure 4F). The technique focuses on the reduction of Cu^2+^ via proteins into a purple complex that can be spectrophotometrically quantified by measuring the absorbance at 562 nm [34]. The total protein concentration of THz treated cells was found to be 28.95 ± 0.06 µg/mL. In comparison, the untreated controls had a relatively higher total protein concentration of 35.02 ± 0.01 µg/mL. Regardless, a statistical analysis did not indicate any statistically significant difference in the protein concentration between the THz-treated cells and the untreated control cells (*p* = 0.574) straight after exposure (Figure 5B). Interestingly, the total protein concentration of the untreated PC 12 cells (Figure 5B) remained higher than that of the THz treated cells over the entire observation period of 7 days. The total protein concentrations were, however, found to be not statistically significant between the two experimental groups when tested on the appropriate days.

**Figure 6:**
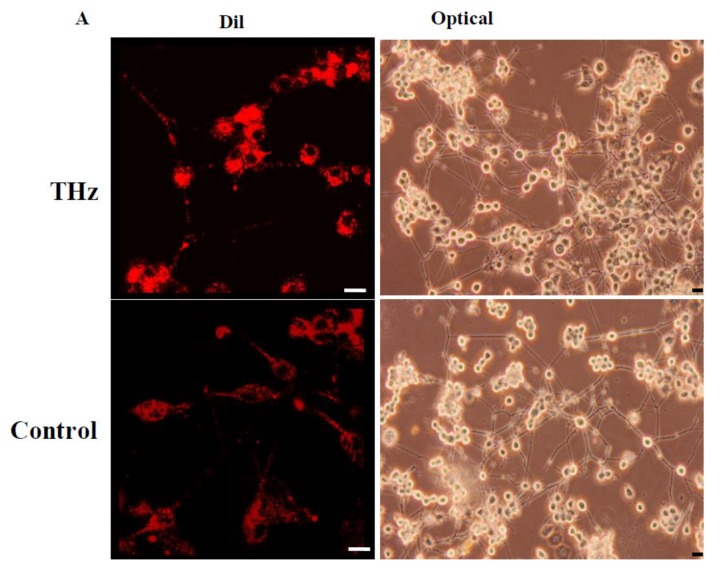

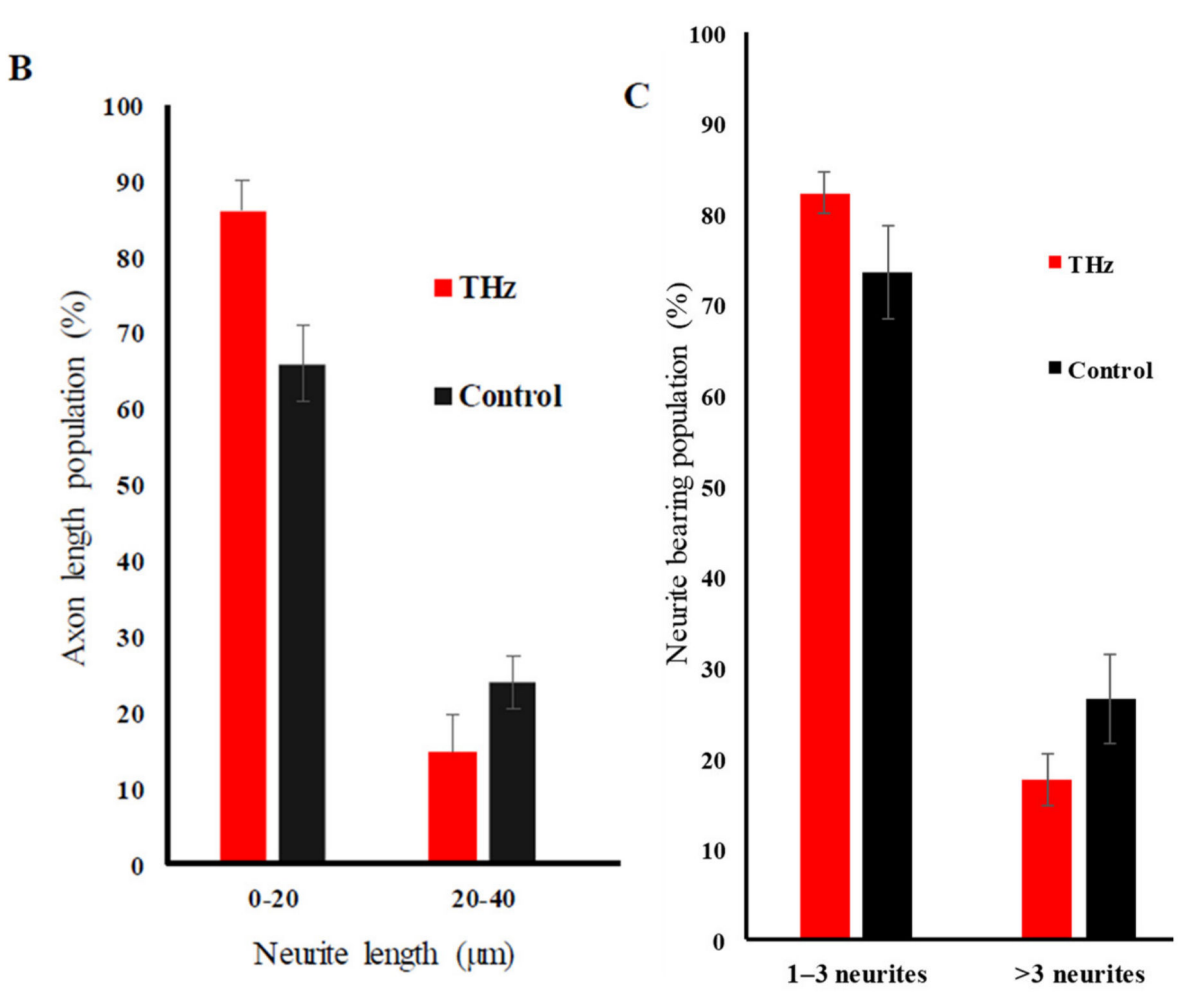
PC 12 cell differentiation at day 7 following exposure to THz radiation. The tissue culture plates were coated with 10 μg/mL of collagen and PC 12 cells seeded at a density of 10^6^ cells/mL. Cells were grown in a low-serum medium with NGF at a concentration of 50 ng/mL. Partial refreshment of the medium was carried out every two days. (**A**) The PC 12 cells were labelled with a lipophilic membrane stain, Dil (1,1′-Dioctadecyl-3,3,3′,3′-Tetramethylindocarbocyanine Perchlorate). No changes were observed among the THz treated PC 12 cells and the untreated control. Scale bar is 10 μm. The phase contrast images were captured on day 7 of the PC 12 cell differentiation. Scale bar is 10 μm. (**B**) Quantification of axon outgrowth. THz treated PC 12 cells and the untreated control was able to undergo neuronal differentiation extending neurites from 0 to >40 μm in diameter with the THz treated sample and the control mainly having extensions from 0–20 μm (*p* = 0.857) and 20–40 μm (*p* = 0.976). (**C**) Quantification of neurite bearing cell population. More than 80% of the THz treated cell population exhibited 1–3 neurite bearings whereas 73% of the control cell population exhibited 1–3 neurite bearings (*p* = 0.855). Even though the THz treated sample demonstrated more neurite bearings in comparison to the control sample, the results were not statistically significant.

PC 12 cells can respond to nerve growth factor (NGF) in serum free or low serum medium by extending neurites and ceasing multiplication [35,36,37]. The effect of THz radiation exposure on PC 12 cell differentiation was further investigated by treating the PC 12 cells with NGF and monitoring the neurite outgrowth for up to 7 days (Figure 6). THz-treated PC 12 cells underwent neuronal differentiation, with 86.17 ± 4.06% of the population extending neurites from 0–20 µm in length, while 14.90 ± 4.88% of the cell population extended neurites of 20–40 µm in length. The untreated control sample consisted of a population of 65.91 ± 5.04% with neurite lengths in the range of 0–20 µm, while 23.86 ± 3.48% had neurites in the length of 20–40 µm. In comparison to the control, the THz-treated cells demonstrated more neurites in the 0–20 µm range on day 7 and less in the range of 20–40 µm.

When analysing the neurite outgrowth, more than 80 ± 2.30% of the THz treated cell population exhibited 1–3 neurite bearings and 73 ± 5.13% of the control cell population exhibited 1–3 neurite bearings. Even though the THz treated sample demonstrated more neurite bearings in comparison to the control sample, the results were not statistically significant (*p* = 0.855).

Considering the findings in this study, and those in the literature, it can be suggested that EMFs of super high frequencies in the THz range induce cell membrane permeabilisation following short exposure times (10 min). These results are in general agreement with those obtained by Fröhlich [38], who concluded that the interactions of THz radiation with cells may arise mediated by the excitation of biological macromolecules or nonlinear/linear resonance mechanisms [1]. Fröhlich also predicted that biological systems are able to support coherent excitations which fall in the 10^9^–10^12^ Hz range [6,38]. The ability of vibrational modes within protein molecules to order and condense into a lowest-frequency vibrational mode in a process similar to Bose–Einstein condensation was demonstrated experimentally using an egg white-derived lysozyme, where exposure to 0.4 THz electromagnetic radiation resulted in a local increase of electron density in a long α-helix motif linked to a slight longitudinal compression of the protein helix [39].

Furthermore, the existing body of literature suggests that the main targets of super high frequency EMF waves in cells are likely to be water molecules, membrane-associated proteins, e.g., proton F_0_F_1_-ATPase bioenergetic enzyme in bacterial cells, and genetic material [40]. When bacterial cells were treated with either low-intensity millimeter waves or electromagnetic fields of extremely high frequencies, strong effects in aqueous suspensions were seen at 50.3, 51.8, 65.5, 64.5, 95 and 105 GHz, and observed biological effects were at least in part attributed to radiation-induced changes in the structure of water molecules present in the surrounding medium [40]. Since water is the main constituent in all biological systems, the energy absorbed might initiate water cluster structuring and subsequent changes (increased or decreased) in chemical activity or the level of hydration in intracellular structures and components, such as membrane-associated proteins [6,40]. Normal functioning of many proteins relies on changes in their conformation, often involving rotational and vibrational frequencies in the THz region. Thus, it is likely that the application of external stimulation at THz frequencies will induce transient conformational changes and temporary changes in the function and level of enzymatic activity of membrane-associated proteins, increasing conductivity and mobility of ions across cell membranes. As such, it is also likely that the mechanism of cell membrane permeabilisation in PC 12 cells is electro-kinetic in nature and is associated with the THz radiation-induced changes in membrane conductivity and ion transport [22,23].

## 3. Materials and Methods

### 3.1. PC 12 Cells Growth Conditions

The pheochromocytoma cell line (PC 12), derived from rat adrenal medulla, was purchased from the American Type Culture Collection (ATCC, Manassas, Virginia, United States of America). PC 12 cells were cultured in complete Gibco™ RPMI medium (Thermo Fisher Scientific, Scoresby, Victoria, Australia) supplemented with 10% Gibco™ horse serum (HS; Thermo Fisher Scientific), 5% Gibco™ foetal bovine serum (FBS; Thermo Fisher Scientific) and 1% Gibco™ penicillin/streptomycin (PS; Thermo Fisher Scientific). Supplements were stored as aliquots at −20 °C. Stock solutions of PC 12 cells were prepared in a medium containing 90% FBS and 10 % DMSO and stored in liquid nitrogen. The cells were maintained at a temperature of 37 °C under a 5% CO_2_ atmosphere in a 95% humidified incubator. The medium was changed every two days and passaged accordingly when the confluence reached 90%.

### 3.2. Sample Preparation and THz Exposure

**Figure 7:**
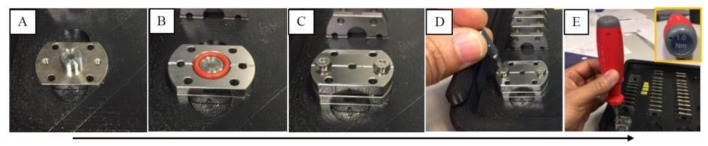
Assembling of the diamond liquid cells (DLC) for the exposure of THz radiation (A–E). (**A**) DLC before loading the sample. (**B**) PC 12 cells were loaded into the DLC with the 300 µm spacer. (**C**) DLC after loading the sample in. (**D**) The three stainless steel components were assembled together. (**E**) The components of the DLC were fitted in place using a screwdriver (Torque 1.0 Nm). After loading the sample, the DLC was viewed under a microscope to confirm the absence of air bubbles.

For this study, a pair of custom-made diamond liquid cells (DLCs) were used to contain the PC 12 samples; the DLCs were labeled “A” and “B”, where “A” was the control and “B” the exposed sample. Each of the DLCs consisted of three stainless steel components (see Figure 7): a 6 mm diameter carbon vapour deposited (CVD) window, 400 µm thick with a 0.5° wedge is glued to two of them, and the third component is a spacer (5–300 µm) that can be changed according to the application; in this application, the 300-µm spacer was used, yielding a volume of 22.4 µL.

**Figure 8:**
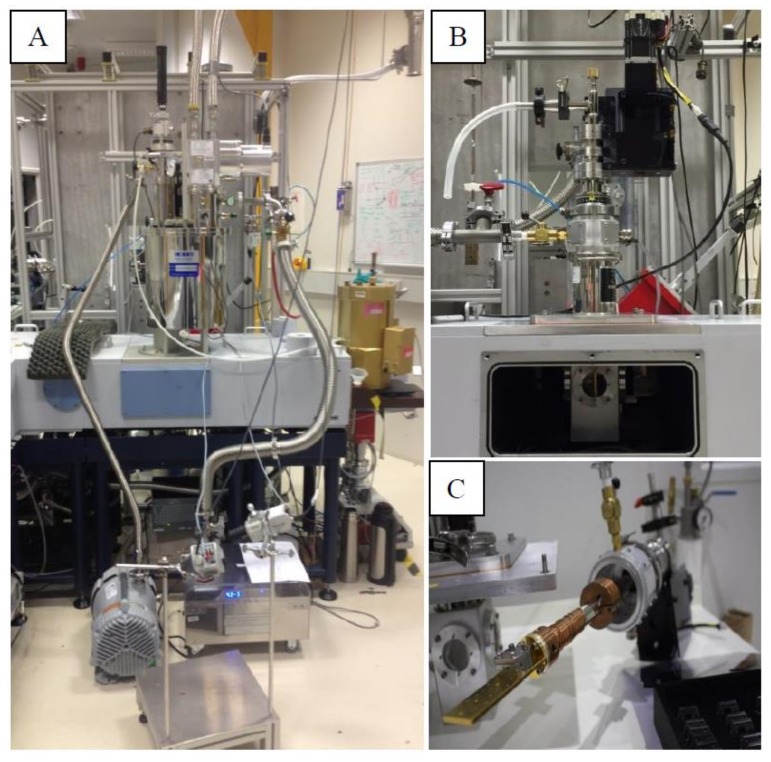
Experimental set up of THz exposure. (**A**) Closed-loop pulse tube cryostat containing the DLC and the (**B**) liquid nitrogen cryostat. (**C**) Sample holder onto which the DLC are mounted on for exposure.

PC 12 cells prepared in growth media were injected into the DLCs, which were then loaded on a Janis ST100 cryostat multi-sample holder (see Figure 8). DLC B was mounted in the middle position of the cryostat where the beam was located while DLC A (the control) was mounted on the top position, and thus the PC 12 cells in this DLC were not exposed to any radiation but were in the same environment as those in DLC B; in this configuration, the PC 12 cells in DLC B were exposed for a period of 10 min to the synchrotron THz radiation.

The Bruker IFS125 spectrometer located in the THz beamline of the Australian Synchrotron (Clayton, Victoria, Australia) was used to expose the PC 12 cells to synchrotron THz radiation; the spectrometer was equipped with a 6 µm Mylar beam splitter. The synchrotron is a broadband source that emits radiation ranging from the THz to hard X-rays; however, the light-energy reaching the sample was limited to 10–650 cm^−1^ (0.3–19.5 THz, λ ≈ 15.38–1000 µm) by the beam splitter. The cell density of the PC 12 cells used for THz exposure was adjusted to 3 × 10^4^ cells per 22.4 µL.

The temperature of the DLC during exposure was recorded repeatedly using an IR-gun (Fluke, Infrared thermometers, Everett, WA, USA) before and at the end of exposure. After the exposure, the DLC was disassembled and the cells were collected for experimental assays.

### 3.3. Cellular Uptake of Silica Nanospheres

Fluorescent silica nanospheres with a diameter of 23.5 ± 0.2 nm (FITC) (Corpuscular, Cold Spring, NY, USA) were used to investigate the permeability of the PC 12 cells immediately after THz radiation exposure. The cell membrane phospholipids were stained using carbocyanine DIL (1,1′-dioctadecyl-3,3,3′,3′-tetramethylindocarbocyanine perchlorate) dye (Thermo Fisher Scientific). The nanospheres were added at a concentration of 10 µg/mL. After a 5-min incubation period, samples were washed twice using PBS and centrifuged at 1300 rpm for 5 min at 25 °C. The procedure was repeated for untreated controls, then the samples were mixed with 10 µL of the FITC nanosphere solution. A 150 µL aliquot of the sample was visualised using a Fluoview FV10i-W inverted microscope (Olympus, Tokyo, Japan).

### 3.4. Permeability Coefficient of THz Radiation Treated PC 12 Cells

The nanosphere uptake following THz radiation exposure was quantified according to the fluorescence intensity generated from silica nanospheres internalised by PC 12 cells using a FLUOstar Omega microplate reader (BMG LABTECH, Cary, NC, USA), following the method that was used previously [25]. The fluorescence readings were due to the silica nanospheres that remained internalized and bound to the PC 12 cells after washing. The mass *m* of a silica nanosphere was determined from the density of silica *ρ* and the volume of a silica nanosphere *V*, related to the radius *r* as *V* = $\frac{4}{3}$π*r*^3^. The average radius of a green nanosphere was 11.75 × 10^−7^ cm (Corpuscular), and hence the volume and mass were estimated to be 6.8 × 10^−18^ cm^3^ and 1.8 × 10^−17^ g, respectively. The mass of a single nanosphere was used to calculate the number of internalized nanospheres. The sample preparation was conducted according to the method used for confocal laser scanning microscopy (CLSM) analysis. The correlation of nanosphere concentration and fluorescence intensity was established using a calibration curve. Standards were prepared using nanosphere concentrations of 0.5, 1, 2, 4, 6, 8, 10, and 12 µg/mL. The cell concentration was adjusted to 60,000 cells per mL after exposure and before recording the level of fluorescence. The concentration of the nanospheres in the THz radiation-exposed suspension was calculated to be 0.05 μg/mL. The concentration of the nanospheres in the THz radiation-exposed suspension was calculated to be 0.05 μg/mL. By dividing the mass of a single nanosphere, the number of internalized nanospheres was 2.7 × 10^12^. Since the cell concentration was adjusted to 60,000 cells/mL, the number of internalized nanospheres per PC 12 cell was calculated to be 4.6 × 10^7^ nanospheres.

CLSM images were used to quantify the nanosphere uptake by counting the number of cells emitting green fluorescence as well as those cells that had not been permeabilized. For each sample, ten different fields of view were analysed. The increase in the degree of permeability is expressed as a percentage.

### 3.5. Transmission Electron Microscopy (TEM) Analysis of Nanosphere Internalisation

After 5 min of incubation in the presence of nanospheres, cell suspensions were pelleted by centrifugation at 1300 rpm for 5 min at 25 °C. The cells were then washed twice with phosphate buffer saline (PBS, 10 mM, pH 7.4) in order to remove any unbound nanospheres. The cell pellet was conditioned with 0.1 M sodium cacodylate buffer (pH 7.4). The cell pellet was then re-suspended in a primary fixative of 4 % paraformaldehyde and 2.5% glutarldehyde in 0.1 M sodium cacodylate buffer overnight at 4 °C and washed thrice in cacodylate buffer for 10 min each. The cells were post-fixed in 1% osmium tetroxide and 1.5% potassium ferrocyanide for 1 h followed by three washes in distilled water for 10 min per wash. The cells were first dehydrated using a graded ethanol series (50%, 70% and 90%) for 15 min, and then further dehydrated twice by passing them through a 100% ethanol series followed by a 100% acetone series for 30 min at room temperature. The cells were then mixed with 1:1 Acetone:Spurr’s resin mixture (Sigma Aldrich, Castle Hill, New South Wales, Australia). After that, the cells were completely exchanged in 100% Spurr’s resin twice for 3 h each.. The resin samples were further polymerised at 70 °C for 48 h. The final block was trimmed, then cut into ultrathin sections (90 nm thickness) using a Leica UltracutUltramicrotome (Leica Microsystems, Wetzlar, Germany) with a diamond knife (Diatome, Pennsylvania, USA). Sections were placed onto 200 mesh copper grids and examined using a JEM 1010 instrument (JEOL). Approximately 40 TEM images were taken at 5000× and 10000× magnifications for sample analysis.

### 3.6. Scanning Electron Microscopy

The cell pellet after THz radiation exposure was re-suspended in a primary fixative of 4% paraformaldehyde and 2.5% glutarldehyde in 0.1 M sodium cacodylate buffer overnight at 4 °C and washed thrice in cacodylate buffer for 10 min each. The cells were post-fixed in 1% osmium tetroxide for 20 mins at room temperature, followed by three washes in distilled water for 10 min each. The cells were first dehydrated using a graded ethanol series (50%, 70% and 90%) for 15 min, then dehydrated further by passing them through a 100% ethanol series twice followed by a 100% acetone series for 30 min at room temperature. The samples were further dehydrated chemically with hexamethyldisilazane (HMDS) and dried overnight. The dried samples were sputter coated with gold using an SPI (Structure Probe, Inc.) sputter coater prior to SEM analysis. The samples were imaged using a FEI Verios(ThermoFisher Scientific, Scoresby, Victoria, Australia) at 5 kV and 100 pA conditions.

### 3.7. Cell Viability

The viability of PC 12 cells was determined using the LIVE/DEAD Viability/Cytotoxicity Kit (Invitrogen, Scoresby, Victoria, Australia). The viability of THz exposed cells and controls was monitored immediately after the treatment and confirmed through three technical replicates. CLSM was used in assessing the number of viable cells; approximately 10 fields of view were analysed per sample type.

### 3.8. Cell Proliferation

Cell proliferation was determined using the CellTiter 96^®^ AQueous One Solution Cell Proliferation Assay (Promega, Sydney Corporate Park, New South Wales, Australia). The assay was performed by adding the tetrazolium compound to the PC 12 cell culture at a 10% ratio of the final volume. This allowed for the reduction of MTS (3-(4,5-dimethylthiazol-2-yl)-5-(3-carboxymethoxyphenyl)-2-(4-sulfophenyl)-2H-tetrazolium) to formazan, which resulted in the formation of a purple-coloured precipitate. The absorbance was recorded at a wavelength of 490 nm after incubation for 90 min at 37 °C using a FLUOstar Omega microplate reader (BMG LABTECH, Mornington, Victoria, Australia).

### 3.9. Protein Concentration of PC 12

The total protein content present in THz radiation treated and untreated cells was determined using the bicinchoninic acid protein (BCA) Assay (Sigma-Aldrich, Castle Hill, New South Wales, Australia). The PC 12 cells were lysed using 150 µL of a protein lysis reagent (Sigma-Aldrich, Castle Hill, New South Wales, Australia) and incubated for 15 min at 25 °C. After incubation, the cells were spun at 1300 rpm for 5 min at 25 °C. 25 µL of the supernatant was then placed on a 96 well plate (Sarstedt, Hildesheim, Lower Saxony, Germany) and 200 µL of BCA reagent (bicinchoninic acid solution and copper (II) sulphate pentahydrate 4%) was added. The sample was then incubated for 30 min at 37 °C and the absorbance was recorded at 562 nm using a FLUOstar Omega micro plate reader (BMG LABTECH).

### 3.10. PC 12 Cell Differentiation

PC 12 cells at a density of 10^5^ cells per mL in Gibco™ 1640 Roswell Park Memorial Institute (RPMI) medium (10% HS, 5% FBS and 1% Pen/Strep) were seeded onto a 10 µg/mL collagen-coated 12-well polystyrene (PS) tissue culture plate according to the manufacturer’s recommended procedure. One day after plating, the full serum culture medium was replaced with a low serum medium supplemented with 50 ng/mL nerve growth factor (mouse recombinant NGF 7S, Sigma-Aldrich, Sydney, NSW, Australia). The culture medium was partially refreshed every two days.

### 3.11. Assessment of Neurite Outgrowth

A phase contrast brightfield inverted Olympus microscope (CKX41, Olympus, Tokyo, Japan) fitted with a Panasonic camera (DMC-GH3) was used to capture images of the differentiating PC 12 cells over a period of 7 days. CLSM was used to record PC 12 differentiation after 7 days. The cell membrane phospholipids were stained using carbocyanine DIL (1,1′-dioctadecyl-3,3,3′,3′-tetramethylindocarbocyanine perchlorate) dye (Thermo Fisher Scientific, Scoresby, Victoria, Australia).

The quantification of the occurrences of neurite extension was carried out using the Neuron J software (Image J plugin; National Institute of Health, Bethesda, MD, USA) by manually tracing the length of the neurites in 10 different fields of view.

The number of neurites per cell were quantified manually in 5–10 different fields of view and the results expressed as a percentage.

### 3.12. Statistical Analysis

Statistical data processing was conducted using the Statistical Package for the Social Sciences, SPSS 24.0 (SPSS, Chicago, IL, USA). The homogeneity of the variances were tested using Levene’s test and statistically significant differences (*p* < 0.05, *p* < 0.01) among the various groups were calculated using a one-way ANOVA analysis [22,41,42], with the independent variables in the study being the two different conditions of treatment.

## 4. Conclusions

In summary, our findings contribute to a more in-depth understanding of how electromagnetic fields of low-energetic intensity operating within microwave and terahertz frequencies interact with cells in the context of environmental exposure, e.g., when these waves are used for communication, remote sensing devices and imaging. Furthermore, our findings suggest that EMFs in the range of THz radiation could be used as a powerful tool to enable a more efficient delivery of genes, nanoparticles and therapeutic drugs, where THz radiation could act as an external stimulus for rapid permeabilisation of the cell membrane while inducing negligible damage due to bulk heating. There is a strong interest in the development of effective yet safe permeabilisation strategies, with currently used techniques including electroporation using an electrical field pulse [43], sonoporation with ultrasound waves [44,45] and photoporation using laser pulses [46].

## References

[1] Bogomazova A.N., Vassina E.M., Goryachkovskaya T.N., Popik V.M., Sokolov A.S., Kolchanov N.A., Lagarkova M.A., Kiselev S.L., Peltek S.E. (2015). No DNA damage response and negligible genome-wide transcriptional changes in human embryonic stem cells exposed to terahertz radiation. Sci. Rep..

[2] Zhao L., Hao Y.H., Peng R.Y. (2014). Advances in the biological effects of terahertz wave radiation. Mil. Med. Res..

[3] Huang Z., Zhang C., Zhang X.-C., Dong L., Cui G., Chang C., Zhao H., Liang J., Zhao X. Research on terahertz properties of rat brain tissue sections during dehydration. Proceedings of the 2017 International Conference on Optical Instruments and Technology: IRMMW-THz Technologies and Applications.

[4] Fan S., He Y., Ung B.S., Pickwell-MacPherson E. (2014). The growth of biomedical terahertz research. J. Phys. D Appl. Phys..

[5] Smye S.W., Chamberlain J.M., Fitzgerald A.J., Berry E. (2001). The interaction between Terahertz radiation and biological tissue. Phys. Med. Biol..

[6] Weightman P. (2012). Prospects for the study of biological systems with high power sources of terahertz radiation. Phys. Biol..

[7] Goryachuk A., Simonova A., Khodzitsky M., Borovkova M., Khamid A. Gastrointestinal cancer diagnostics by terahertz time domain spectroscopy. Proceedings of the 2017 IEEE International Symposium on Medical Measurements and Applications (MeMeA).

[8] Bowman T., El-Shenawee M., Campbell L.K. (2016). Terahertz transmission vs. reflection imaging and model-based characterization for excised breast carcinomas. Biomed. Opt. Express.

[9] Wilmink G.J., Rivest B.D., Roth C.C., Ibey B.L., Payne J.A., Cundin L.X., Grundt J.E., Peralta X., Mixon D.G., Roach W.P. (2011). In vitro investigation of the biological effects associated with human dermal fibroblasts exposed to 2.52 THz radiation. Lasers Surg. Med..

[10] Am O.B., Amit T., Youdim M.B.H. (2004). Contrasting neuroprotective and neurotoxic actions of respective metabolites of anti-Parkinson drugs rasagiline and selegiline. Neurosci. Lett..

[11] Subramanian T., Emerich D.F., Bakay R.A.E., Hoffman J.M., Goodman M.M., Shoup T.M., Miller G.W., Levey A.I., Hubert G.W., Batchelor S. (1997). Polymer-encapsulated PC-12 cells demonstrate high-affinity uptake of dopamine in vitro and ^18^F-DOPA uptake and metabolism after intracerebral implantation in nonhuman primates. Cell Transplant..

[12] Hoop M., Chen X.Z., Ferrari A., Mushtaq F., Ghazaryan G., Tervoort T., Poulikakos D., Nelson B., Pane S. (2017). Ultrasound-mediated piezoelectric differentiation of neuron-like PC12 cells on PVDF membranes. Sci. Rep..

[13] Clothier R.H., Bourne N. (2003). Effects of THz Exposure on Human Primary Keratinocyte Differentiation and Viability. J. Biol. Phys..

[14] Fischer B.M., Walther M., Jepsen P.U. (2002). Far-infrared vibrational modes of DNA components studied by terahertz time-domain spectroscopy. Phys. Med. Biol..

[15] Alexandrov B.S., Gelev V., Bishop A.R., Usheva A., Rasmussen K.Ø. (2010). DNA Breathing Dynamics in the Presence of a Terahertz Field. Phys. Lett. A.

[16] Bock J., Fukuyo Y., Kang S., Phipps M.L., Alexandrov L.B., Rasmussen K.Ø., Bishop A.R., Rosen E.D., Martinez J.S., Chen H.-T. (2011). Mammalian Stem Cells Reprogramming in Response to Terahertz Radiation. PLoS ONE.

[17] Ol’shevskaia I., Kozlov A.S., Petrov A.K., Zapara T.A., Ratushniak A.S. (2009). Influence of terahertz (submillimeter) laser radiation on neurons in vitro. Zhurnal Vysshei Nervnoi Deiatelnosti Imeni I P Pavlova.

[18] Lindenbaum M.H., Carbonetto S., Grosveld F., Flavell D., Mushynski W.E. (1988). Transcriptional and post-transcriptional effects of nerve growth factor on expression of the three neurofilament subunits in PC-12 cells. J. Biol. Chem..

[19] Refolo L.M., Salton S.R.J., Anderson J.P., Mehta P., Robakis N.K. (1989). Nerve and epidermal growth factors induce the release of the Alzheimer Amyloid precursor from PC 12 cell cultures. Biochem. Biophys. Res. Commun..

[20] Avedisian C.T., Cavicchi R.E., McEuen P.L., Zhou X. (2009). Nanoparticles for cancer treatment: Role of heat transfer. Ann. N. Y. Acad. Sci..

[21] Selvi B.R., Jagadeesan D., Suma B.S., Nagashankar G., Arif M.K., Balasubramanyam M.E., Kundu T.K. (2008). Intrinsically Flourescent Carbon Nanospheres as a Nuclear Targeting Vector: Delivery of Membrane-Impermeable Molecule to Modulate Gene Expression In Vivo. Nano Lett..

[22] Perera P.G.T., Nguyen T.H.P., Dekiwadia C., Wandiyanto J.V., Sbarski I., Bazaka O., Bazaka K., Crawford R.J., Croft R.J., Ivanova E.P. (2018). Exposure to high-frequency electromagnetic field triggers rapid uptake of large nanosphere clusters by pheochromocytoma cells. Int. J. Nanomed..

[23] Nguyen T.H., Shamis Y., Croft R.J., Wood A., McIntosh R.L., Crawford R.J., Ivanova E.P. THz radiation studies on biological systems at the ENEA FEL facility. Infrared Phys. Technol..

[24] Shamis Y., Taube A., Mitik-Dineva N., Croft R., Crawford R.J., Ivanova E.P. (2007). Permeability changes induced by 130 GHz pulsed radiation on cationic liposomes loaded with carbonic anhydrase. Bioelectromagnetics.

[25] Nguyen T.H., Pham V.T., Nguyen S.H., Baulin V., Croft R.J., Phillips B., Crawford R.J., Ivanova E.P. (2015). 18 GHz electromagnetic field induces permeability of Gram-positive COCCI. Sci. Rep..

[26] Tinevez J.Y., Schulze U., Salbreux G., Roensch J., Joanny J.F., Paluch E. (2009). Role of cortical tension in bleb growth. PNAS.

[27] Paluch E.K., Raz E. (2013). The role and regulation of blebs in cell migration. Curr. Opin. Cell Biol..

[28] Antonopoulos A., Eisenbrandt H., Obe G. (1997). Effects of high frequency electromagentic fields on human lymphocytes in vitro. Mutat. Res..

[29] Malich G., Markovic B., Winder C. (1997). The sensitivity and specificity of the MTS tetrazolium assay for detecting the in vitro cytotoxicity of 20 chemicals using human cell lines. Toxicology.

[30] Lobner D. (2000). Comparison of the LDH and MTT assays for quantifying cell death: Validity for neuronal apoptosis?. J. Neurosc. Methods.

[31] Shamis Y., Traub A., Croft R., Crawford R., Ivanova E.P. (2012). Influence of 18GHz microwave radiation on the enzymatic activity of *Escherichia coli* lactate dehydrogenase and cytochrome C oxidase. J. Phys. Sci. App..

[32] Valbonesi P., Franzellitti S., Bersani F., Contin A., Fabbri E. (2016). Activity and expression of acetylcholinesterase in PC12 cells exposed to intermittent 1.8 GHz 217-GSM mobile phone signal. Int. J. Radiat. Biol..

[33] Nishiki T., Narumiya S., Morii N., Yamamoto M., Fujiwara M., Kamata Y., Sakaguchi G., Kozaki S. (1990). ADP-Ribosylation of the Rho/Rac proteins induces growth inhibition, neurite outgrowth and acetylcholine esterase in cultured PC 12 cells. Biochem. Biophys. Res. Commun..

[34] Smith P.K., Krohn R.I., Hermanson G.T., Mallia A.K., Gartner F.H., Provenzano M.D., Fujimoto E.K., Goeke N.M., Olson B.J., Klenk D.C. (1985). Measurement of Protein Using Bicinchoninic Acid. Anal. Biochem..

[35] Greene L.A. (1978). Nerve Growth Factor Prevents The Death And Stimulates The Neuronal Differentiation Of Clonal PC 12 Pheochromocytoma Cells In Serum-Free Medium. J. Cell Biol..

[36] Wandiyanto J.V., Linklater D., Tharushi Perera P.G., Orlowska A., Truong V.K., Thissen H., Ghanaati S., Baulin V., Crawford R.J., Juodkazis S. (2018). Pheochromocytoma (PC12) Cell Response on Mechanobactericidal Titanium Surfaces. Materials.

[37] Orlowska A., Perera P.T., Al Kobaisi M., Dias A., Nguyen H.K.D., Ghanaati S., Baulin V., Crawford R.J., Ivanova E.P. (2017). The Effect of Coatings and Nerve Growth Factor on Attachment and Differentiation of Pheochromocytoma Cells. Materials.

[38] Fröhlich H. (1968). Long-range coherence and energy storage in biological systems. Int. J. Quantum Chem..

[39] Lundholm I.V., Rodilla H., Wahlgren W.Y., Duelli A., Bourenkov G., Vukusic J., Friedman R., Stake J., Schneider T., Katona G. (2015). Terahertz radiation induces non-thermal structural changes associated with Fröhlich condensation in a protein crystal. Struct. Dyn..

[40] Soghomonyan D., Trchounian K., Trchounian A. (2016). Millimeter waves or extremely high frequency electromagnetic fields in the environment: What are their effects on bacteria?. Appl. Microbiol. Biotechnol..

[41] Ogra Y., Tejima A., Hatakeyama N., Shiraiwa M., Wu S., Ishikawa T., Yawata A., Anan Y., Suzuki N. (2016). Changes in intracellular copper concentration and copper-regulating gene expression after PC12 differentiation into neurons. Sci. Rep..

[42] Lazarov-Spiegler O., Solomon A.S., Zeev-Brann A.B., Hirschberg D.L., Lavie V., Schwartz M. (1996). Transplantation of activated macrophages overcomes central nervous system regrowth failure. FASEB J..

[43] Sadiq A.A., Zaltum M.A.M., Mamman H.B., Adon M.N., Othman N.B., Dalimin M.N., Jamil M.M.A. An overview: Investigation of electroporation and sonoporation Techniques. Proceedings of the 2015 2nd International Conference on Biomedical Engineering (ICoBE).

[44] Yu H., Xu L. (2014). Cell experimental studies on sonoporation: State of the art and remaining problems. J. Control. Release.

[45] Ohta S., Suzuki K., Ogino Y., Miyagawa S., Murashima A., Matsumaru D., Yamada G. (2008). Gene transduction by sonoporation. Dev. Growth Differ..

[46] Banavath H.N., Allam S.R., Valathati S.S., Sharan A., Rajasekaran B. (2018). Femtosecond laser pulse assisted photoporation for drug delivery in Chronic myelogenous leukemia cells. J. Photochem. Photobiol. B.

